# Disulfiram Treatment for Alcohol Abuse

**DOI:** 10.1111/adb.70061

**Published:** 2025-07-02

**Authors:** Colin Brewer, Emmanuel Streel

**Affiliations:** ^1^ No affiliation; ^2^ University of Brussels (ULB) Brussels Belgium

Dear Editor,

As the authors or co‐authors of numerous contributions to the disulfiram (DSF) literature since the 1980s and the co‐authors of the first textbook devoted to DSF treatment for some 40 years [1], we read Schallenberg et al.'s paper [2] with interest and some anticipatory excitement. Their positive findings are consistent with several other DSF studies in which consumption was carefully supervised. Diligent supervision is crucial for the success of DSF treatment, and it is something that we have stressed since the early 1980s [3], including the first study that examined the techniques that some patients use to evade or sabotage it [4]. We were therefore surprised that they do not directly cite these papers and earlier ones. As long ago as the 1960s, Bourne et al. published the first such study [5], which recorded surprisingly good outcomes in a group of recurrent ‘skid‐row’ alcoholic offenders, not normally regarded as promising candidates for treatment. Azrin et al. published one of the first controlled studies of supervised versus unsupervised DSF in 1982 [6]. Haynes echoed Bourne et al.'s achievements with a 13‐fold reduction in alcohol‐related offending [7], whereas Sereny et al. found that outpatient treatment with clinic‐supervised DSF was widely accepted when it was made a required condition of continuing treatment after two failures [8].

Equally surprising is their failure to reference either of the two papers which not only reviewed the effectiveness of long‐term supervised DSF but also suggested the mechanisms that made supervised DSF so effective [9, 10], even when DSF was discontinued after a year or two of good progress, as in the OLITA study that they cited. They include exposure and response‐prevention, a well‐validated treatment for repetitive and/or phobic behaviour. It embodies and facilitates the repeated practice, learning and consolidation of new and more appropriate habits in real‐life environments, as opposed to the artificial and protected environments of residential and outpatient clinics.

They also make the common mistake of describing DSF as ‘aversive’, even though in most studies, the majority of patients never risk drinking while taking it. It is therefore more correctly described as a ‘deterrent’ drug, and it deters drinking in the same way that speed cameras deter speeding without most drivers having to be fined first. A similar deterrent effect is seen in studies of ‘instant justice’ programmes for repeat driving while intoxicated (DWI) offenders, in which failure to produce a negative breathalyser test every morning and evening at their local police station results in instant, unappealable overnight imprisonment (an alcohol‐sensitive electronic ankle tag is an alternative) [11]. Despite many of them clearly qualifying for a diagnosis of alcohol use disorder, ‘over 99% of tests are negative and 53% of offenders never test positive. A further 19% test positive only once and 11% do so twice’ [12]. The authors specifically state that ‘[The reduction in offending] seems to be largely a deterrent effect’.

We feel that the missing references, with their important practical and theoretical implications for DSF treatment, should not be overlooked. As it happens, Dr (now Professor) Peter Bourne is still very much alive.

## Data Availability

The data that support the findings of this study are available on request from the corresponding author. The data are not publicly available due to privacy or ethical restrictions.
